# MicroRNA-1252-5p, regulated by Myb, inhibits invasion and epithelial-mesenchymal transition of pancreatic cancer cells by targeting NEDD9

**DOI:** 10.18632/aging.203344

**Published:** 2021-07-27

**Authors:** Yuzheng Xue, Tielong Wu, Yingyue Sheng, Yao Zhong, Benshun Hu, Chuanqing Bao

**Affiliations:** 1 Department of Gastroenterology, Affiliated Hospital of Jiangnan University, Wuxi, Jiangsu Province, China; 2 Department of Hepatobiliary Surgery, Affiliated Hospital of Jiangnan University, Wuxi, Jiangsu Province, China; 3 Department of Gastrointestinal Surgery, Affiliated Hospital of Jiangnan University, Wuxi, Jiangsu Province, China

**Keywords:** miR-1252-5p, epithelial-mesenchymal transition, pancreatic cancer, NEDD9, Myb

## Abstract

MicroRNAs (miRNAs) are known to be involved in the development and progression of pancreatic cancer (PAC). The expression levels and roles of miR-1252-5p in PAC remain unclear. Quantitative real-time PCR and *in situ* hybridization were used to detect miR-1252-5p expression in PAC cells and human tissues. We studied the gain and loss of function of miR-1252-5p in the PAC cell lines *in vitro* and *in vivo*. The direct targets of miR-1252-5p were analyzed using public databases and a dual-luciferase reporter assay. Expression levels of miR-1252-5p are downregulated in PAC cell lines and tissue samples, and its expression is negatively associated with adverse clinical features and poor prognosis. *In vitro* and *in vivo* experiments show that miR-1252-5p overexpression inhibits the proliferation, migration, invasion, and epithelial-mesenchymal transition of PAC cells, and miR-1252-5p knockdown enhances these biological behaviors. MiR-1252-5p negatively regulates neural precursor cell expressed, developmentally downregulated 9 (NEDD9) by directly binding its 3'- untranslated region. Further mechanism research revealed that the SRC/STAT3 pathway is involved in miR-1252-5p/NEDD9 mediation of PAC's biological behaviors. We also verified that Myb inhibited miR-1252-5p by directly binding at its promoter. MiR-1252-5p may act as a tumor-suppressing miRNA in PAC and may be a potential therapeutic target for PAC patients.

## INTRODUCTION

In the United States, pancreatic cancer (PAC) is the fourth most common cause of cancer-related death, with an estimated 40,560 deaths in 2015 [1]. Despite remarkable advances in diagnosis and therapy, patients' long-term survival with PAC remains unsatisfactory due to high rates of distant metastasis [2]. It is, therefore, essential to study the underlying mechanisms of PAC aggressiveness and invasion.

MicroRNAs (miRNAs) are a group of endogenous evolutionarily conserved non-coding small RNAs that can regulate the expression of target mRNA via post-transcriptionally binding its 3′-untranslated region (UTR) [3]. It has been demonstrated that aberrantly expressed miRNAs contribute to cancer growth, apoptosis, distant metastasis, and drug-resistance in PAC [4, 5]. In humans, miR-1252-5p, previously named miR-1252, is located on 12q21.2, and has been reported to be involved in the progression of various cancer types, including non-small cell lung cancer(NSCLC) [6, 7] and head and neck cancers [8]. However, the expression, biological roles, and the underlying molecular mechanisms of miR-1252-5p in PAC remain fully characterized.

It is well established that epithelial-to-mesenchymal transition (EMT) is linked to the proliferation and invasion of various cancer types, including PAC [9]. During EMT, epithelial cells are transformed to have an invasive mesenchymal phenotype [10] and exhibit changes in expression of EMT markers: downregulated E-cadherin (E-cad) and upregulated Vimentin (Vim) and N-cadherin (N-cad). Additional transcription factors, including Twist, the Snail/Slug family, and ZEB1/ZEB2, act as molecular switches in the EMT process [10]. EMT-regulating miRNAs, including miR-200c and miR-21, have been increasingly reported in association with cancer [11]. Mostly, miRNAs negatively regulate EMT, with only a small number of them (such as miR-21) inducing EMT. However, the association between miR-1252-5p and EMT in PAC has not been investigated.

This study examined miR-1252-5p expression levels and clinical significance in human PAC tumor tissues (TT) and non-cancerous adjacent tissues (NAT). In human PAC cell lines, miR-1252-5p targets the 3′-UTR of the mRNA of neural precursor cell expressed, developmentally downregulated 9 (NEDD9). Studies have observed that NEDD9 is upregulated and may promote the invasion and metastasis of many types of human malignancy, including PAC [12, 13]. We also demonstrate that the SRC/STAT3 pathway may be involved in the biological function of miR-1252-5p targeting of NEDD9 in PAC.

## MATERIALS AND METHODS

### Ethics and reagents

The study protocol was performed according to the principles of the Declaration of Helsinki and the ethics committee of the Affiliated Hospital of Jiangnan University, Wuxi, Jiangsu Province, China. Informed consent was obtained from all patients.

The following primary antibodies were purchased from Abcam (Cambridge, England): anti-E-cad, anti-Vim, anti-Snail, anti-N-cad, anti-Twist, anti-PCNA, anti-Bax, anti-Bcl-2, anti-ZEB1, anti-NEDD9, anti-Myb, and anti-GAPDH. The following primary antibodies were purchased from Cell Signaling Technology (Beverly, MA, USA): anti-t-SRC, anti-p-SRC, anti-t-STAT3, and anti-p-STAT3. PP1, an SRC inhibitor, was purchased from Selleck Chemicals (Houston, TX, USA).

### Study population and follow-up

PAC tumor tissue samples and matched NATs were collected from 102 patients who underwent surgery in the Department of Hepatobiliary Surgery at the Affiliated Hospital of Jiangnan University (Wuxi, Jiangsu Province, China) between January 2014 and December 2015. Primary carcinomas were assessed according to the 7th edition of the American Joint Committee on Cancer (AJCC) staging system [14] and the World Health Organization classification [15]. None of the patients had received neoadjuvant therapy before surgical resection.

### *In situ* hybridization, immunohistochemistry, and scoring

For *in situ* hybridization, slides were hybridized overnight at 60° C with a miRCURY DIG-labelled locked nucleic acid (LNA)-based probe specific for miR-1252-5p according to the manufacturer’s protocol (Exiqon, Vedbaek, Denmark). For immunohistochemistry, these slides were incubated with antibodies against E-cad and Vim using the DAKO Envision system (DAKO, Carpinteria, CA, USA), as described previously [16].

The staining results were scored by the multiply of staining intensity (0,1, 2, and 3 points scored for none, weak, intermediate and strong staining, respectively) and percentage of positive cells (0, 1, 2, and 3 scored for <10%, 10% to 50% and >50% positive tumor cells, respectively). Thus, the expression of miR-1252-5p, E-cad, Vim, and NEDD9 were scored overall as 0, 1, 2, 3, 4, 6, or 9. Expression was defined as “low” if the final score was < 4 points and as “high” if it was > 4 points. Scoring was performed by two independent pathologists who were blinded to the clinicopathological characteristics of the samples. Kappa statistics were employed as a measurement of agreement between the two pathologists.

### Cell culture

A total of 5 human PAC cell lines, ASPC-1, CAPAN-2, Panc-1, SW1990, and BxPC-3, and a normal human pancreatic epithelium cell line (HPNE) were purchased from the American Type Culture Collection. Cells were cultured in standard Dulbecco’s modified Eagle’s medium (DMEM, Corning, USA) supplemented with 10% fetal bovine serum (FBS), 100 units/ml penicillin and 100 μg/ml streptomycin and grown in a humidified incubator with 5% CO_2_ at 37° C.

### Cell transfection

MiR-1252-5p inhibitor, mimic, and their negative controls (NC), anti-miR-NC and miR-NC, were designed and synthesized by GenePharma (Shanghai, China). The overexpression plasmid for human NEDD9 (pcDNA3.1-NEDD9) and Myb (pcDNA3.1-Myb) and the negative controls (empty vector [EV]) were synthesized by GenePharma (Shanghai, China). Specific small hairpin RNA (shRNA) targeting NEDD9 (ShNEDD9), Myb (shMyb), and the negative control (sh-NC) were also purchased from GenePharma (Shanghai, China). Briefly, 1 × 10^5^ cells (35 mm^3^ well plate) were seeded. The plasmid vectors and siRNAs were transfected into PAC cells using Lipofectamine 2000 Reagent (Invitrogen, Carlsbad, CA, USA) following the manufacturer’s instructions at a final concentration of 5 nM for 24 h. Each transfection experiment was independently repeated at least in triplicates. The knockdown and overexpression efficiencies were evaluated by quantitative real-time PCR (qRT-PCR).

### qRT-PCR

All tissues were confirmed by pathological analysis and immediately frozen in liquid nitrogen and stored at -80° C. According to the manufacturer's instructions, TRIzol reagent (Invitrogen, Carlsbad, CA, USA) was used to extract total RNA from TTs, NATs, and PAC cell lines. qRT-PCR was performed using a SYBR® Premix Ex Taq kit (Takara Bio Inc., Shiga, Japan) and a 96-well real-time PCR system (Bio-Rad Inc., Hercules, CA, USA). GAPDH or U6 snRNA was used as a loading control. The primers for miR-1252-5p were purchased from Sangon Inc., (Shanghai, China), and the details of the primers for GAPDH, NEDD9, E-cad, N-cad, ZEB1, Twist, Snail, and Vim are listed in Supplementary Table 1. Relative gene expression was calculated from the qRT-PCR data using the 2 ^−ΔΔCT^ method.

### Western blotting

Whole protein was extracted from tissues and cells using RIPA buffer (Roche, Basel, Switzerland). A BCA Protein Assay Kit (Thermo Fisher Scientific) was used to determine protein concentration. An equal amount of protein was separated by 10% SDS-PAGE and then transferred into PVDF membranes (Millipore, Billerica, MA, USA). The membrane was probed with a HRP-conjugated secondary antibody. The protein bands were visualized using an electrochemiluminescence reagent (ECL; Pierce Chemical Co., Rockford, IL, USA). GAPDH was used as a loading control, and quantitative analysis was performed using Image J software. Other detailed methods were shown in Supplementary Material.

### Availability of data and materials

The datasets used and/or analyzed during the current study are available from the corresponding author on reasonable request.

### Ethics approval and consent to participate

All procedures performed in the present study involving human specimens were approved by the Ethics Committee of the affiliated hospital of Jiangnan University, Wuxi, Jiangsu Province. All patients provided written informed consent prior to their inclusion within the study. The study was conducted according to the principles outlined in the Declaration of Helsinki.

## RESULTS

### MiR-1252-5p expression was downregulated in PAC specimens and cell lines

**Figure 1:**
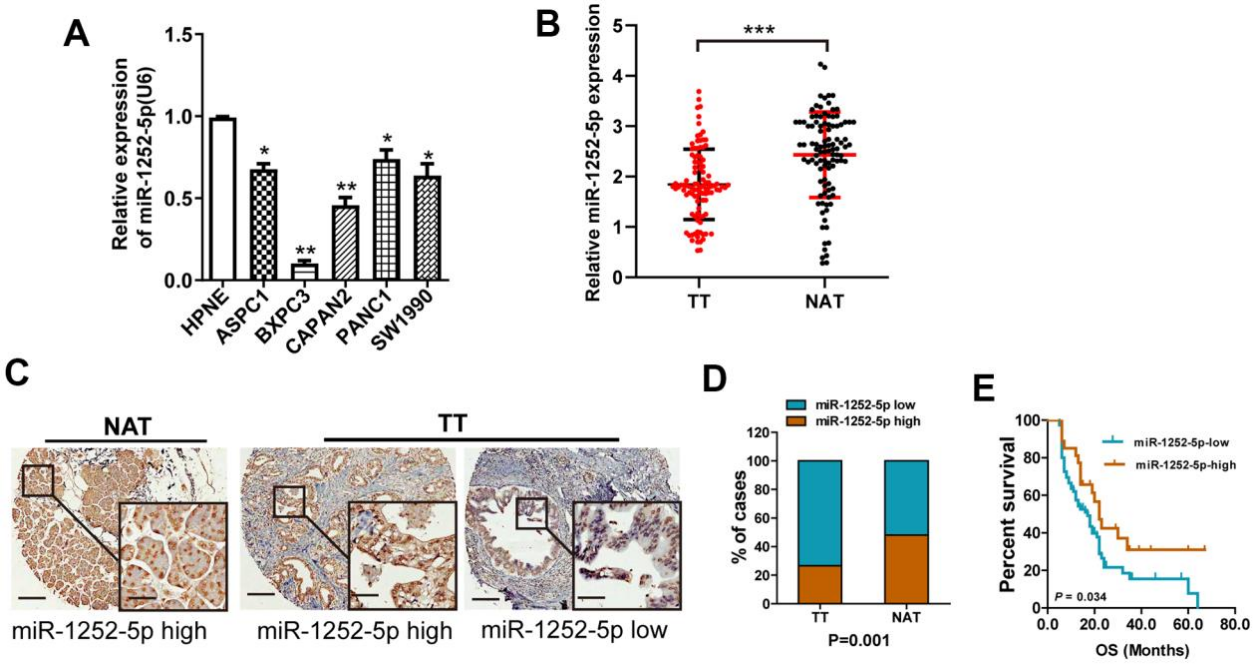
**MiR-1252-5p expression in PAC cell lines and tissue samples.** (**A**) qRT-PCR assay analyses of miR-1252-5p expression in five human PAC cell lines and HPNE. (**B**) qRT-PCR assay analysis of miR-1252-5p expression in 102 matched fresh human TT and NAT pancreas samples. Data were obtained using the 2^-ΔΔCT^ method and were normalized to U6 levels. (**C**, **D**) *In situ* hybridization assay and staining score analyses were used to determine miR-1252-5p expression levels in 102 matched human TT and NAT pancreas samples. Scale bar, 200μm; scale bar for inserts, 40μm. (**E**) Kaplan-Meier survival curves showed association of miR-1252-5p expression with overall survival in patients with PAC (*P* = 0.034, log-rank test). PAC, pancreatic cancer; TT, tumor tissues; NAT, non-cancerous adjacent tissues. *, P<0.05; **P < 0.01; ***, *P* < 0.001.

Using qRT-PCR, we first assessed miR-1252-5p expression levels in cell lines (ASPC-1, CAPAN-2, SW1990, Panc-1, BxPC 3, and HPNE cells). MiR-1252-5p expression was significantly reduced in the 5 PAC cell lines than HPNE cells (Figure 1A). Moreover, expression of miR-1252-5p in TT from 102 patients was markedly lower than that in matched NAT (*P* < 0.001; Figure 1B). Expression of miR-1252-5p was also assessed in a tissue microarray (TMA) containing 102 human TT and matched NAT pancreatic specimens using ISH. According to the calculated staining score, the expression levels of miR-1252-5p were significantly lower in TT compared to NAT (*P* < 0.001; Figure 1C, 1D).

To determine the role of miR-1252-5p in PAC progression, we evaluated the relationship between miR-1252-5p expression and clinicopathological characteristics. We observed that low expression of miR-1252-5p was significantly correlated with node invasion and high histologic grade, but not with tumor diameter, neural invasion, or T stage (Supplementary Figure 1A–1E). Additionally, Kaplan-Meier curves indicated that PAC patients with low miR-1252-5p expression had a notably decreased OS (*P* = 0.034; Figure 1E). Univariate and multivariate cox regression survival analysis indicated that downregulated miR-1252-5p expression was an independent prognostic indicator of poor OS (hazard ratio [HR] = 1.59; 95% confidence interval [CI]: 1.01-2.50; *P* = 0.046; Table 1). These results suggested that miR-1252-5p might be correlated with tumor progression in PAC.

**Table 1:** Prognostic factors in Cox’s proportional hazards model.

**Parameters**	**Median OS (95%CI) (months)**	**Univariate analysis**			**Multivariate analysis**	
		**HR (95%CI)**	**P**		**HR (95%CI)**	**P**
**Tumor size, cm**						
< 3.5	15.7(11.7-18.9)	1.0	0.061		1.0	0.075
≥3.5	12.2(10.1-13.4)	1.62(0.98-2.69)			1.33(0.97-1.82)	
**T stage**						
T1-2	18.8(12.9-24.3)	1.0	0.032		1.0	0.038
T3	12.9(11.5-14.3)	1.81(1.05-3.12)			1.29(1.01-1.65)	
**N stage**						
N0	14.8(12.3-18.5)	1.0	0.143			
N1	11.5(9.1-13.9)	1.47(0.88-2.44)				
**Grade**						
Poor	12.5(9.6-14.3)	1.0	0.353			
Good+ moderate	15.7(13.1-18.0)	0.77(0.45-1.33)				
**Neural invasion**						
Negative	15.9(13.3-18.9)	1.0	0.006		1.0	0.029
Positive	11.1(10.2-12.8)	2.06(1.23-3.46)			1.67(1.05-2.05)	
**Vascular invasion**						
Negative	13.4(12.3-14.5)	1.0	0.766			
Positive	12.6(10.1-15.3)	0.86(0.31-2.36)				
**MiR-1252-5p score**						
High	19.2(14.0-23.6)	1.0	0.051		1.0	0.046
Low	12.2(10.9-13.5)	1.79(1.00-3.23)			1.59(1.01-2.50)	

Note: Tumor classification and stage were referred to the 7th edition of UICC (2009) on cancer staging system.

### MiR-1252-5p inhibited proliferation, migration, and invasion in PAC cells

**Figure 2:**
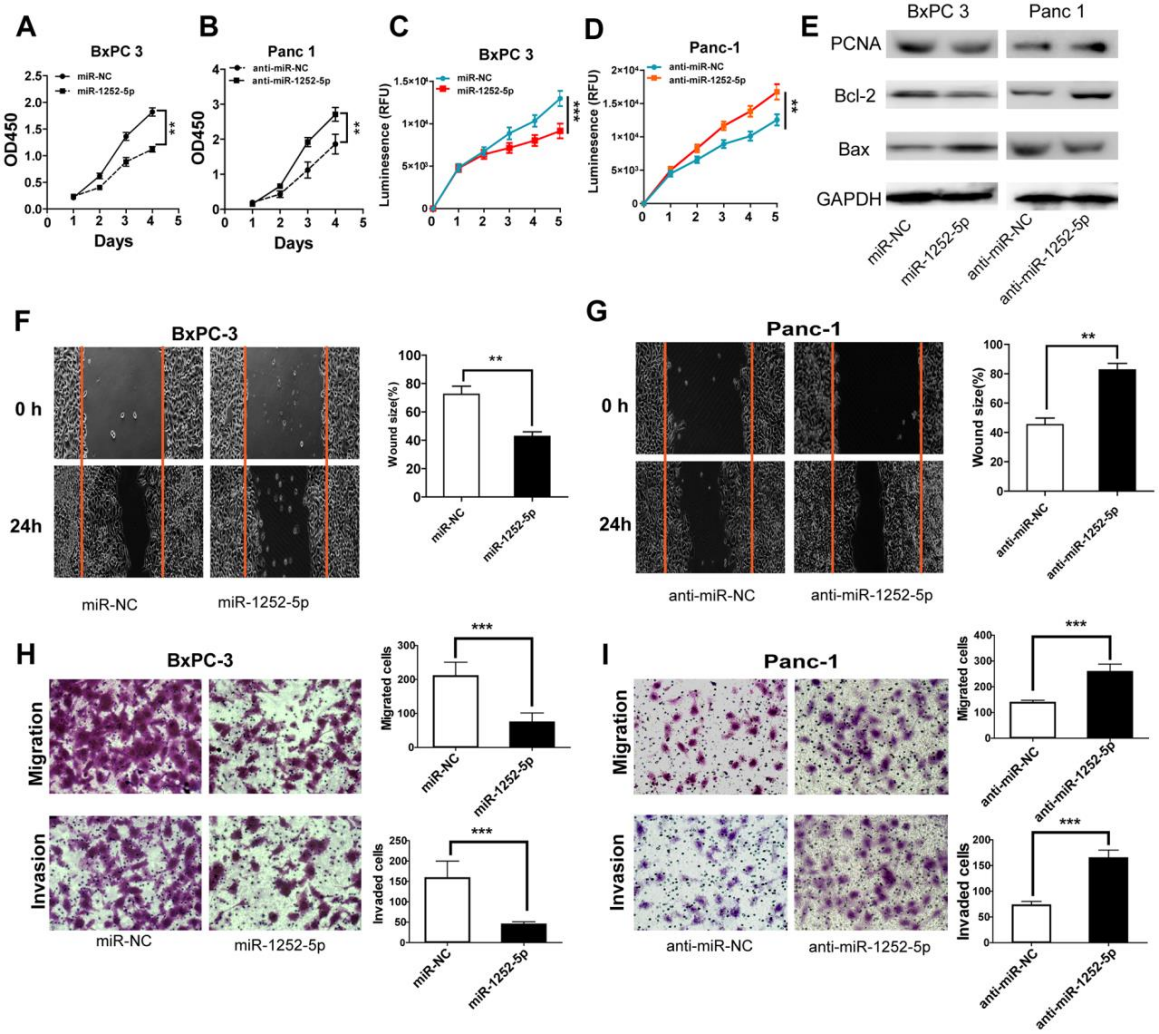
**MiR-1252-5p inhibited the proliferation, migration and invasion of PAC cells.** (**A**–**D**) MTT and CellTiter-Glo luminescent cell viability assays were conducted to detect cell proliferation after transfection with corresponding miRNA vectors in PAC cells. (**E**) Western blot assays were conducted to examine markers of cell proliferation and apoptosis (PCNA, Bcl-2 and Bax) after transfection with corresponding miRNA vectors in PAC cells. (**F**–**I**) Wound healing and Transwell (without or with Matrigel) assays analysis of cell migratory and invasive ability after transfection with corresponding miRNA vectors in PAC cells. **P < 0.01; ***, *P* < 0.001.

To explore the biological role of miR-1252-5p in PAC, we conducted gain- and loss-of-function studies in BxPC3 and Panc-1 cells, respectively. It was found that miR-1252-5p was effectively upregulated in BxPC3 cells and downregulated in Panc-1 cells (*P* < 0.001; Supplementary Figure 2A, 2B). MTT and CellTiter-Glo luminescent cell viability assays indicated significantly inhibited cell proliferation in the miRNA mimic group compared with the NC group, while significantly enhanced cell proliferation was observed in the inhibitor group (Figure 2A–2D). Western blotting assay of cell proliferation and apoptosis markers showed that miR-1252-5p overexpression significantly inhibited expression of PCNA and Bcl-2, but increased expression of Bax. MiR-1252-5p knockdown led to the opposite change of these markers (Figure 2E and Supplementary Figure 2C, 2D). Moreover, wound closure and transwell assays also confirmed that miR-1252-5p overexpression significantly reduced, whereas knockdown of miR-1252-5p promoted PAC cells' migratory and invasive ability, respectively (*P* < 0.05; Figure 2H, 2I).

### MiR-1252-5p inhibited EMT process in PAC

**Figure 3:**
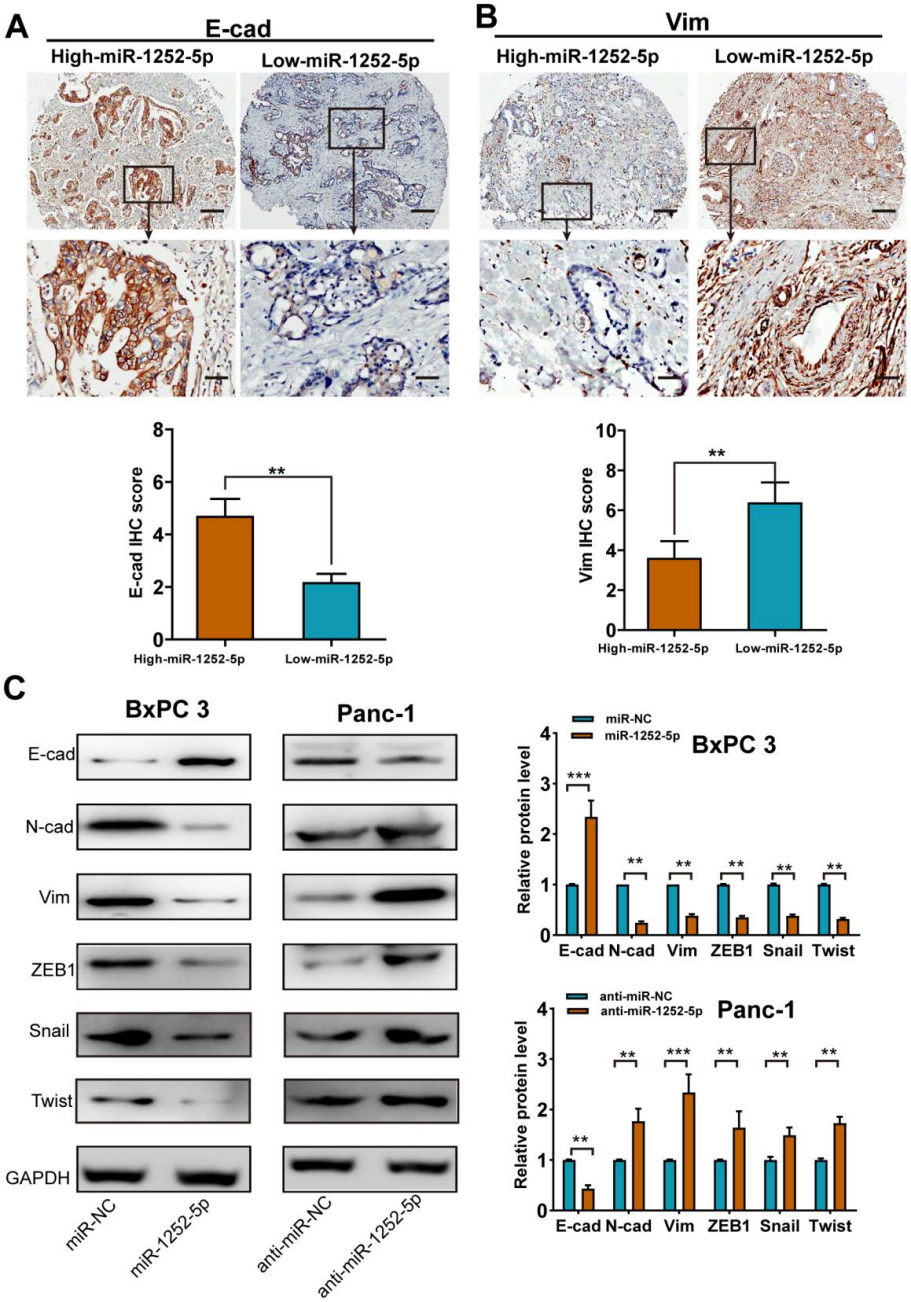
**MiR-1252-5p inhibited the EMT process of PAC cells.** (**A**, **B**) Representative immunohistochemical staining and histograms for E-cad (**A**) and Vim (**B**) expression were showed and compared between high- and low-miR-1252-5p expressing PAC tissues. Scale bar, 200μm for upper row and 40μm for lower row. (**C**) Western blot analysis of EMT markers (E-cad, N-cad, Vim, ZEB1, Snail and Twist) after transfection with corresponding miRNA vectors in PAC cells. GAPDH served as a loading control. E-cad, E-cadherin; N-cad, N-cadherin; Vim, Vimentin. **P < 0.01; ***, *P* < 0.001.

We then explored whether miR-1252-5p modulated PAC progression via regulating the EMT process. In human PAC tissues, miR-1252-5p expression was found to positively related to E-cad expression (*P* < 0.01; Figure 3A), but inversely related to Vim expression (*P* < 0.01; Figure 3B). Western blotting assays revealed that miR-1252-5p overexpression enhanced E-cad expression and decreased N-cad, Vim, ZEB1, Twist, and Snail expression in BxPC 3 cells, while anti-miR-1252-5p induced the opposite in Panc-1 cells (Figure 3C). Moreover, we determined EMT marker expression by qRT-PCR and observed similar results (*P* < 0.01; Supplementary Figure 3A, 3B). Collectively, these results implicate that miR-1252-5p suppressed EMT process in PAC.

### NEDD9 was a direct target of miR-1252-5p in PAC

**Figure 4:**
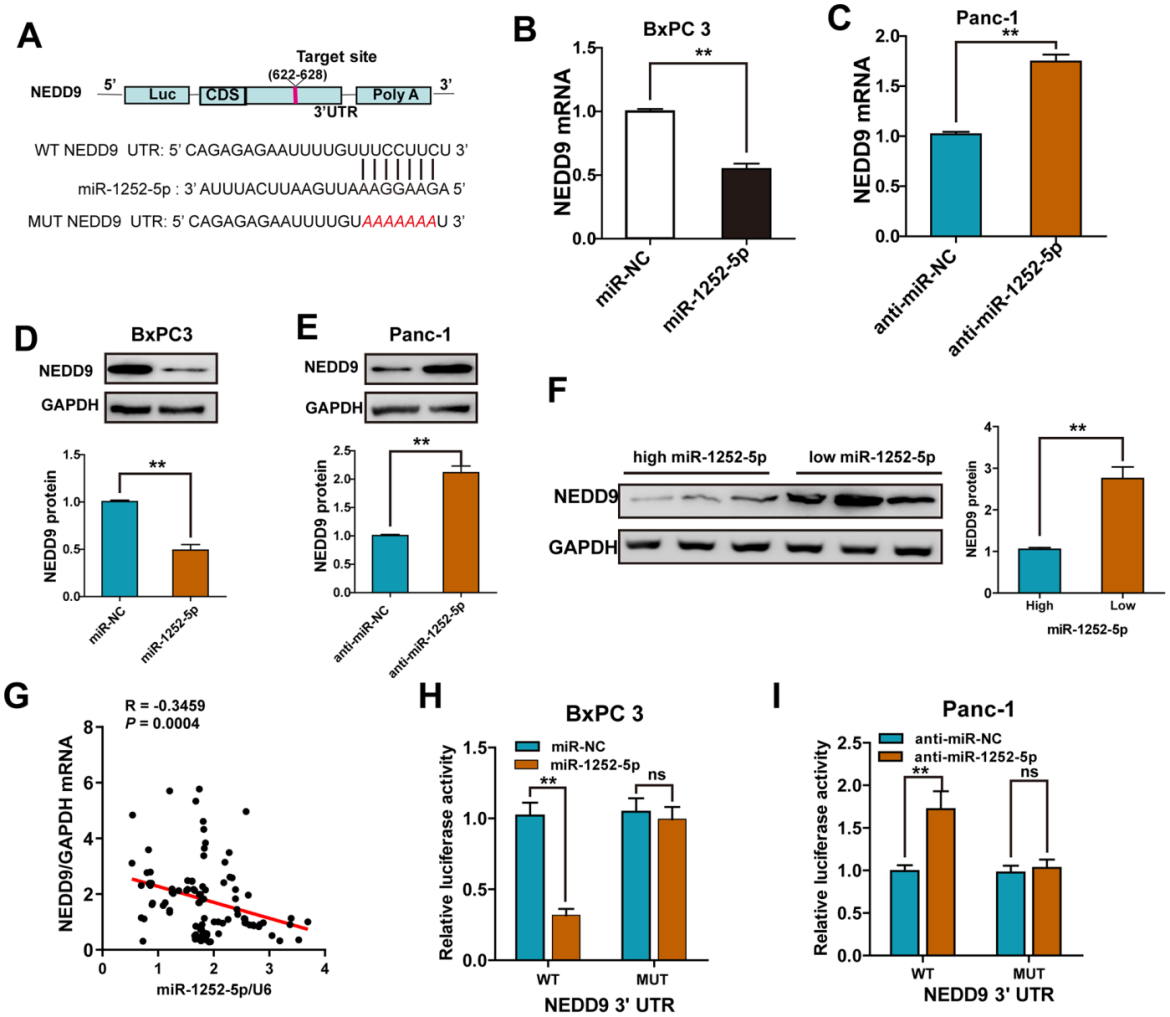
**NEDD9 was a direct target of miR-1252-5p in PAC cells.** (**A**) miR-1252-5p and its putative binding sequences in the 3′-UTR of NEDD9. (**B**–**E**) qRT-PCR and western blot analysis of NEDD9 mRNA and protein expression after transfection with corresponding miRNA vectors in PAC cells. (**F**) Western blot assays of NEDD9 protein expression in human PAC tissues with high- and low-miR-1252-5p expressing. (**G**) An inverse correlation between miR-1252-5p and NEDD9 mRNA levels was observed in human PAC tissues (n = 18). (**H**, **I**) Luciferase activity was detected after co-transfection with corresponding miRNA vectors and luciferase vectors in PAC cells. NEDD9, neural precursor cell expressed, developmentally downregulated 9; PAC, pancreatic cancer; WT, wild-type; MUT, mutant-type; UTR, untranslated region. **P < 0.01; ***, *P* < 0.001, ns, not significant.

Using a miRNA target algorithm (Starbase 3.0), we then predicted potential targets and observed conserved putative miR-1252-5p binding sites at the 3′-UTR of NEDD9 (Figure 4A). We selected NEDD9 as a potential target for further experiments because our previous study [13] found that NEDD9 was overexpressed in PAC tumor tissues. High NEDD9 expression was significantly correlated with advanced clinical stage, lymph node metastasis, and poor prognosis in PAC patients. By qRT-PCR and western blotting, we found that overexpression or knockdown of miR-1252-5p markedly decreased or enhanced the mRNA and protein expression levels of NEDD9 in PAC cells, respectively (Figure 4B–4E). Moreover, we found that the protein expression of NEDD9 in human PAC tissues expressing high levels of miR-1252-5p was significantly lower than those expressing low levels (Figure 4F). An inverse correlation between miR-1252-5p and NEDD9 mRNA expression was validated in human PAC tissues by Spearman’s correlation analysis (*P* = 0.0004; Figure 4G). Next, overexpression or knockdown of miR-1252-5p significantly decreased or enhanced the luciferase activity of wild-type (WT) NEDD9 3′-UTR, respectively. Altering miR-1252-5p expression did not substantially change the luciferase activity of mutant (MUT) NEDD9 3′-UTR cells (Figure 4H, 4I). These data indicate that NEDD9 was a downstream target of miR-1252-5p in PAC.

### NEDD9 mediated the biological functions of miR-1252-5p in PAC cells

**Figure 5:**
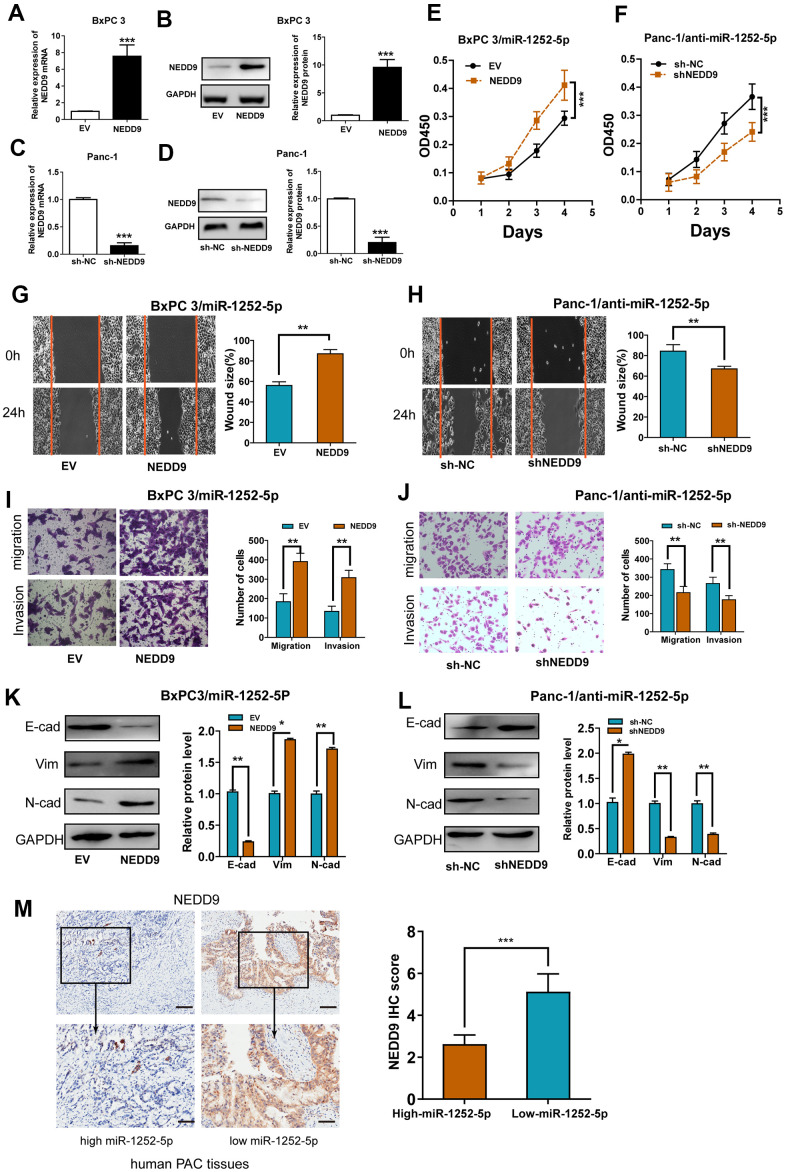
**Modulation of NEDD9 partially restored miR-1252-5p-mediated cellular functions in PAC.** (**A**–**D**) qRT-PCR and western blot analysis of NEDD9 mRNA and protein expression after transfection with corresponding vectors in PAC cells. (**E**, **F**) MTT assays were conducted to detect cell proliferation after transfection with corresponding vectors in PAC cells. (**G**–**J**) Wound healing and Transwell (without or with Matrigel) assays analysis of cell migratory and invasive ability after transfection with corresponding miRNA vectors in PAC cells. (**K**, **L**) Western blotting assays were conducted to detect NEDD9 and EMT markers (E-cad, N-cad, Vim) after transfection with corresponding vectors in PAC cells. (**M**) IHC assay analysis of the association between miR-1252-5p expression and NEDD9 expression in human PAC tissues. Scale bar, 100μm for upper row and 40μm for lower row. NEDD9, neural precursor cell expressed, developmentally downregulated 9; E-cad, E-cadherin; N-cad, N-cadherin; Vim, Vimentin. *P < 0.05; **P < 0.01; ***, *P* < 0.001.

To confirm the biological role of NEDD9 in the effects of miR-1252-5p on PAC, we successfully upregulated or knocked down NEDD9 expression via transfecting pcDNA3.1-NEDD9 (Figure 5A, 5B) or shRNA targeting NEDD9 (Figure 5C, 5D). NEDD9 overexpression promoted PAC cell proliferation, migration, and invasion, while silencing NEDD9 resulted in significantly reduced malignant behaviors (Supplementary Figure 4A–4H). Furthermore, rescue experiments showed that up-regulation of NEDD9 restored the miR-1252-5p overexpression-inhibited proliferation, migration, invasion, and EMT process in BxPC3 cells. Simultaneously, knockdown of NEDD9 blocked the miR-1252-5p inhibitor-enhanced biological effects on Panc-1 cells (Figure 5E–5L). We also explored the association between miR-1252-5p expression and NEDD9 expression in human PAC tissues and found that miR-1252-5p expression was inversely related to NEDD9 expression (*P* < 0.01; Figure 5M). These results supported the hypothesis that NEDD9 acted as a functional mediator of miR-1252-5p in PAC.

### MiR-1252-5p inhibited tumor xenograft growth of PAC *in vivo*

**Figure 6:**
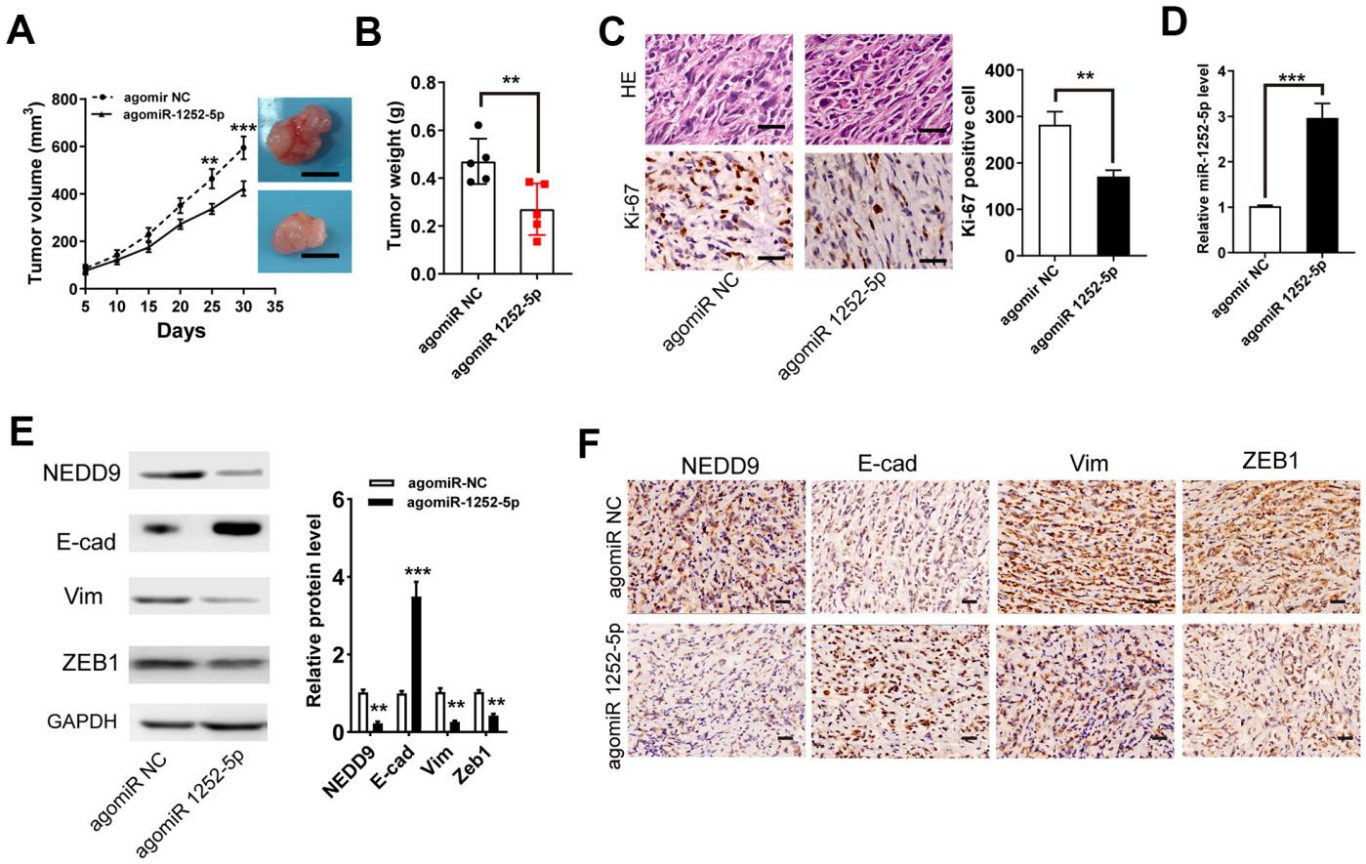
**MiR-1252-5p inhibited tumor xenograft growth of PAC *in vivo*.** (**A**, **B**) Tumor volumes and weights were represented. Scale bar, 1.0 cm. (**C**) The tumor sections were subjected to H&E staining and IHC staining using antibodies against ki-67. Scale bar, 100μm. (**D**) qRT-PCR assay analysis of the expression of miR-1252-5p in the xenograft tumors. (**E**, **F**) Western blot and IHC staining assay analysis of protein expression of NEDD9, E-cad, Vim, and ZEB1 in the xenograft tumors. Scale bar, 200μm. Data are presented as mean ± SD from triplicate experiments. IHC, immunohistochemistry. **P < 0.01; ***P < 0.001.

To validate the functions of miR-1252-5p in the growth of PAC *in vivo*, we performed a mouse xenograft experiment. As exhibited in Figure 6A, 6B, agomiR 1252-5p treatment significantly suppressed tumor volume (Figure 6A) and weight (Figure 6B). IHC staining of Ki-67 clarified that agomiR 1252-5p treatment markedly decreased tumor proliferation (Figure 6C). Furthermore, qRT-PCR assay verified enhanced levels of miR-1252-5p expression in tumors with agomiR 1252-5p-treatment compared with control tumors (Figure 6D). Importantly, western blot and IHC staining assay analysis confirmed that agomiR 1252-5p treatment markedly decreased protein expression of NEDD9, E-cad, Vim, and ZEB1 in the xenograft tumors (Figure 6E, 6F).

### The SRC/STAT3 pathway was involved in the biological roles of miR-1252-5p/NEDD9 in PAC

**Figure 7:**
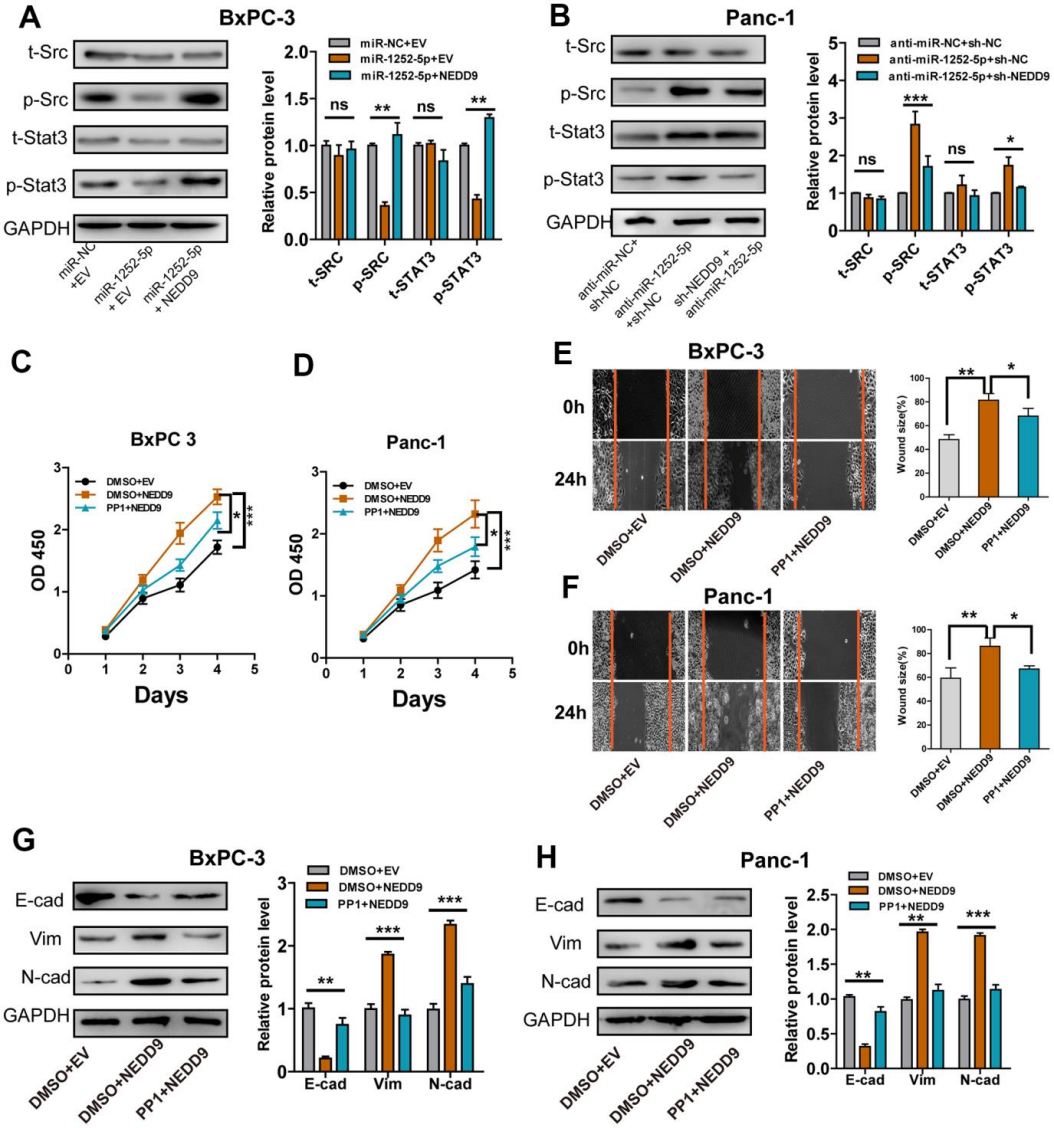
**SRC/STAT3 signaling was essential for the biological function of miR-1252-5p/NEDD9 in PAC.** (**A**, **B**) Western blot analysis of the activation of SRC/STAT3 signaling protein after transfection with corresponding vectors in PAC cells. C-H, MTT, wound healing and western blotting assays analysis of cell proliferation (**C**, **D**), migration (**E**, **F**) and EMT process (**G**, **H**) after transfection with corresponding vectors or treatment with PP1 in PAC cells. NEDD9, neural precursor cell expressed, developmentally downregulated 9; NC, negative control. E-cad, E-cadherin; N-cad, N-cadherin; Vim, Vimentin. ns, not significant; *P < 0.05; **P < 0.01; ***P < 0.001.

It has been shown that NEDD9 acted through SRC and STAT3 to promote invasion in melanoma, cervical cancer, and ovarian cancer [17–19]. To investigate whether this pathway is involved in the biological roles of miR-1252-5p/NEDD9 axis in PAC development, we examined these proteins' phosphorylation states. NEDD9 upregulation at least partially rescued the levels of phosphorylated SRC and STAT3 inhibited by miR-1252-5p overexpression in BxPC-3 cells, while modulating NEDD9 expression did not alter the levels of both total proteins (Figure 7A). Furthermore, inhibition of miR-1252-5p induced activation of SRC/STAT3 signaling, and NEDD9 knockdown at least partially inhibited these effects (Figure 7B). Besides, MTT and wound healing assays showed that NEDD9 stimulated proliferation and migration of PAC cells (Figure 7C–7F), which was significantly impeded in both cell lines incubated with PP1, an inhibitor of SRC. Western blotting assays showed that NEDD9-induced EMT relied on SRC activity in BxPC-3 (Figure 7G) and Panc-1 cells (Figure 7H). Collectively, these data indicated that the SRC/STAT3 pathway was involved in the biological function of miR-1252-5p/NEDD9 in PAC.

### Myb inhibited miR-1252-5p expression through binding its promoter

**Figure 8:**
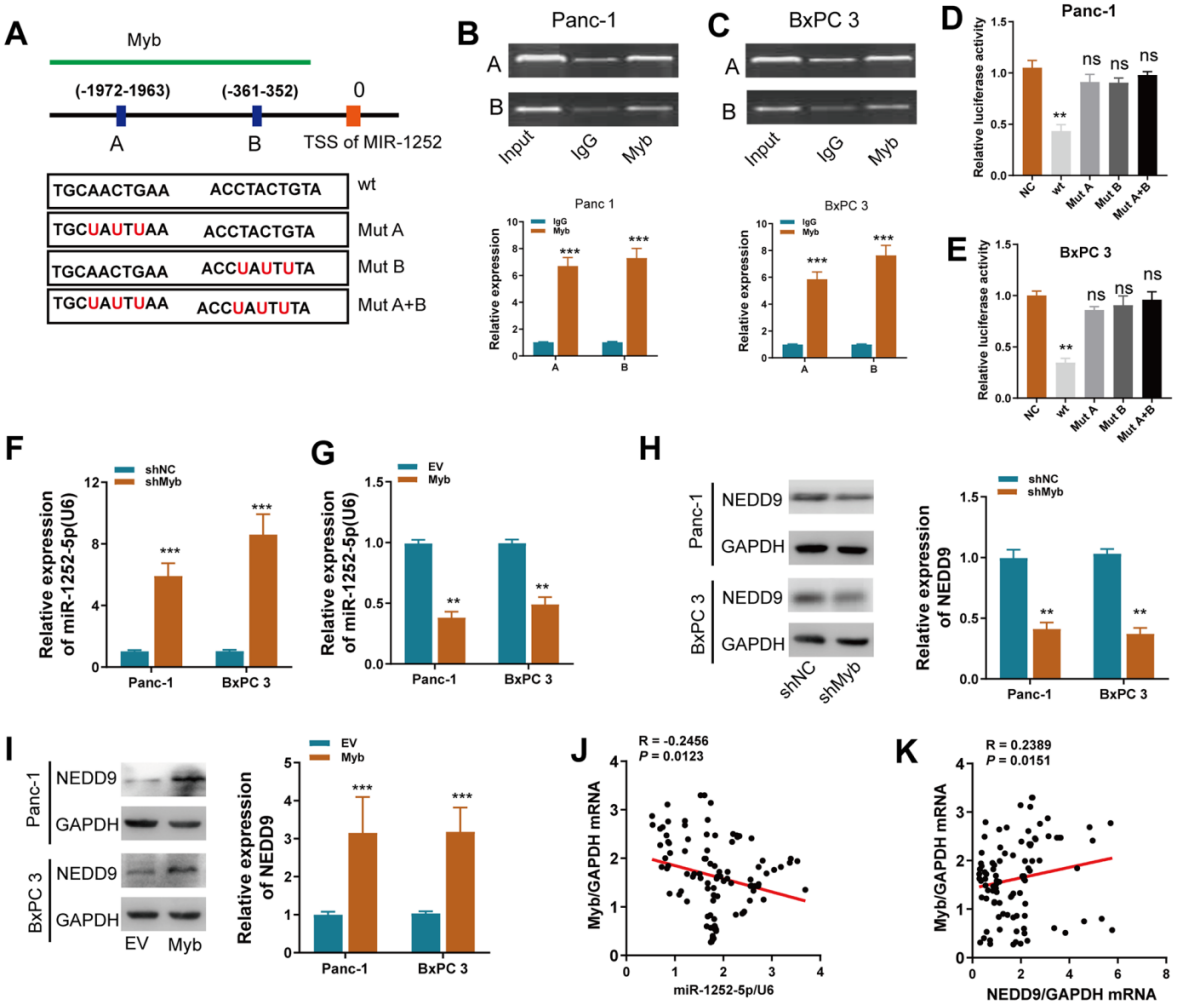
**Myb regulated miR-1252-5p/NEDD9 axis at the transcriptional level in PAC.** (**A**) Schematic of the promoter regions of miR-1252 with potential Myb binding sites and the wild-type (wt) and mutant-type (mut) sites. (**B**, **C**) ChiP assay analyses of chromatin-bound antibody against Myb in PAC cells. IgG antibody was used as a negative control. (**D**, **E**) Dual-luciferase reporter assay in PAC cells. The relative luciferase activity was normalized to the Renilla luciferase activity. (**F**, **G**) qRT-PCR assay analyses of miR-1252-5p expression in PAC cells after transfection with the indicated vectors. (**H**, **I**) Western blot assay analyses of NEDD9 expression in PAC cells after transfection with the indicated vectors. J-K, The correlation of Myb mRNA with miR-1252-5p (**J**) or NEDD9 mRNA (**K**) in human PAC tissues (n = 102). CDS, coding sequence; PAC, pancreatic cancer; ns, not significant. Data are presented as mean ± SD from triplicate experiments. **P < 0.01; ***, *P* < 0.001.

We used the JASPER bioinformatics software program to search a 2 kb region upstream of the transcription start site (TSS) of miR-1252. Two Myb-binding motifs from 1963 to −1972 and −352 to −361 were identified, named A and B (Figure 8A), and ChIP assay confirmed that Myb protein was indeed recruited to these two binding sites in both cells (Figure 8B, 8C). Next, reduced luciferase activity in the wt miR-1252 promoter was observed after overexpression of Myb in both cells (Figure 8D, 8E). These effects were not observed when the A and/or B sites were mutated. We found that ectopic expression of Myb inhibited, while silencing of Myb increased, miR-1252-5p expression in PAC cells (Figure 8F, 8G). On the contrary, expression of NEDD9 protein in PAC cells was decreased or increased after expression of Myb was silenced or upregulated, respectively (Figure 8H, 8I). Furthermore, Myb expression was inversely correlated with miR-1252-5p expression, but positively with NEDD9 expression in human PAC tissues (Figure 8J, 8K).

### MiR-1252-5p inhibited Myb-induced cell biological behaviors in PAC cells

**Figure 9:**
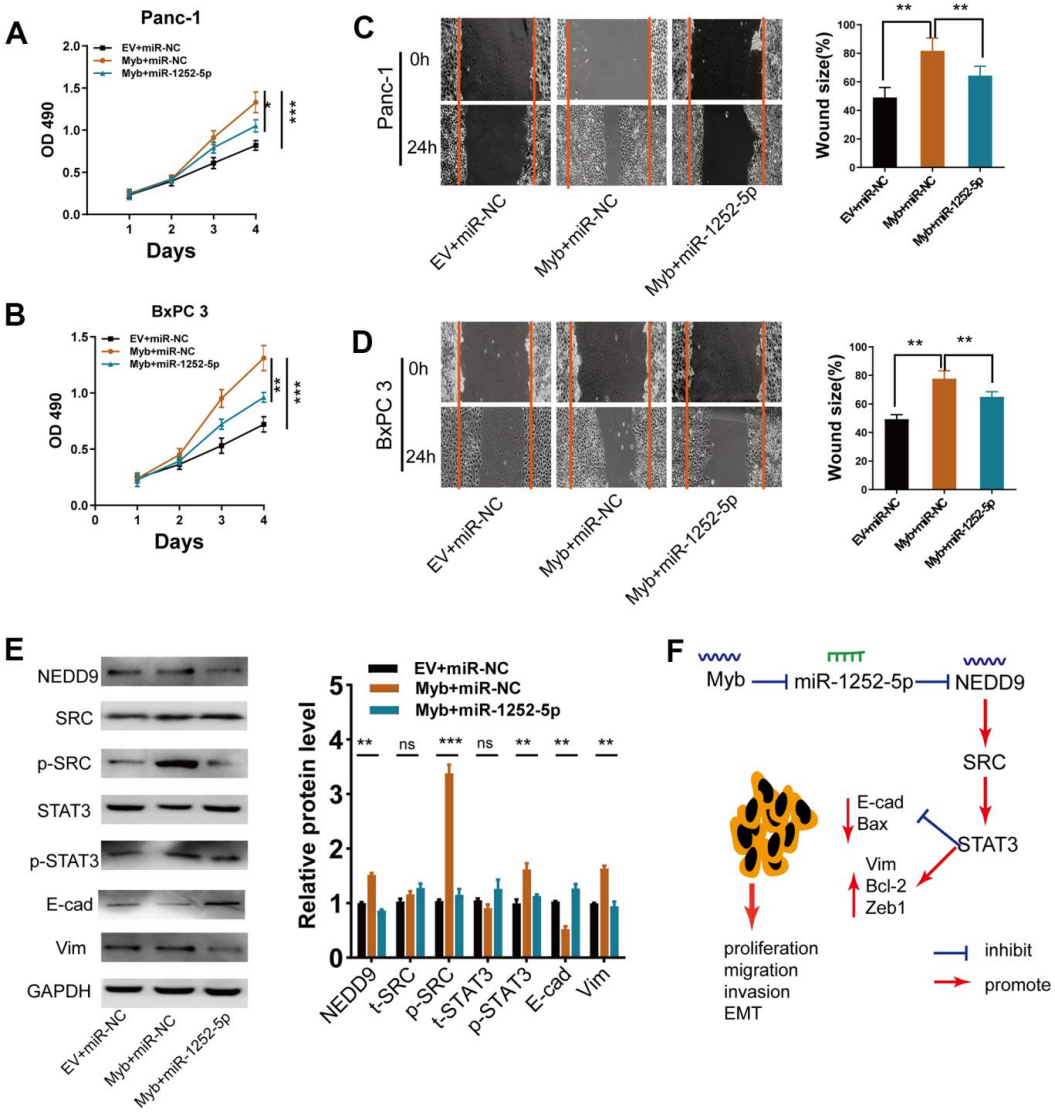
**MiR-1252-5p overexpression reversed Myb-induced cellular biological behaviors.** (**A**, **B**) MTT assay analyses of cell proliferation in PAC cells after transfection with the indicated vectors. (**C**, **D**) Wound healing assay analyses of cell migration in PAC cells after transfection with the indicated vectors. (**E**) Western blot assay analyses of expression of NEDD9, EMT markers, SRC/STAT3 protein in PAC cells after transfection with the indicated vectors. (**F**) A working model depicts the mechanism of transcriptional regulation of Myb on miR-1252-5p targeting NEDD9 and the subsequent activation of SRC/STAT3 signal pathways contributed to PAC progression. E-cad, E-cadherin; Vim, Vimentin. Data are presented as mean ± SD from triplicate experiments. *, *P*<0.05; **P < 0.01; ***, *P* < 0.001.

We then explored if miR-1252-5p played a role in the Myb-induced phenotypes in PAC cells. Myb overexpression significantly increased PAC cell growth (Figure 9A, 9B), migration (Figure 9C, 9D), while miR-1252-5p overexpression reversed these effects. Furthermore, ectopic Myb significantly enhanced NEDD9 expression, the activation of SRC/STAT3 pathway, and the EMT process, while miR-1252-5p overexpression significantly attenuated these effects (Figure 9E). These findings indicated that miR-1252-5p counteracted Myb- induced PAC growth, EMT process, and the subsequent activation of SRC/STAT3 via targeting NEDD9.

## DISCUSSION

Although significant progress has been made in cancer therapy, PAC still develops resistance to current standard treatments, resulting in a poor prognosis [2]. Evidence suggests that miRNAs serve significant roles in regulating genes during the development of various cancer types, including PAC [20]. In this study, we observed significantly downregulated expression of miR-1252-5p in PAC tissues and cell lines for the first time. We demonstrated that miR-1252-5p plays a suppressive oncogene role in PAC by directly targeting NEDD9 to inhibit activation of SRC/STAT3 signaling, consequently impeding PAC progression. Specifically, miR-1252-5p expression was inhibited by Myb at the transcriptional level. These data indicate that miR-1252-5p may be helpful as a biomarker for predicting prognosis and may be a potential therapeutic target for PAC.

MiR-1252-5p was downregulated in human PAC cell lines and tissues compared with normal pancreatic cells and NAT. Furthermore, low miR-1252-5p expression was closely associated with node invasion and high histologic grade, but not with T stage, neural invasion, or tumor diameter. Low expression of miR-1252-5p was identified by multivariate Cox regression analysis as an independent predictor of poor OS for PAC patients who underwent tumor resection. Intriguingly, gain- and loss-of-function experiments revealed that overexpression of miR-1252-5p inhibited the proliferation, migration, invasion, and EMT of PAC cells, while knockdown of miR-1252-5p enhanced these aggressive behaviors. EMT, characterized by acquiring a migratory and invasive mesenchymal phenotype, is a major contributor to cancer-cell aggressiveness and metastasis [21]. EMT has also been demonstrated to be regulated by miRNAs such as miR-202 [22] and miR-200c [23]. In the present study, expression of epithelial (E-cad) and mesenchymal markers (Vim, N-cad, ZEB1) were increased or decreased in PAC cells, when miR-1252-5p expression was upregulated. On the contrary, the opposite was achieved when miR-1252-5p expression was inhibited.

The present study's significant finding is that miR-1252-5p functions as a tumor-suppressive miRNA in human PAC by inhibiting cell mobility, invasiveness, and proliferation via directly inhibiting its downstream target, NEDD9. Firstly, miR-1252-5p negatively regulated NEDD9 expression at the mRNA and protein levels in PAC cells. Secondly, miR-1252-5p expression was inversely correlated with the expression of NEDD9 in PAC tissues. Finally, we identified the complementary sequence of miR-1252-5p in the 3’UTR of NEDD9 mRNA. The overexpression or knockdown of miR-1252-5p altered the luciferase activity with the wide type but not the mutant type 3’UTR of NEDD9.

NEDD9 is a member of the non-catalytic Crk-associated substrate (CAS) family of scaffolding proteins, which functions as a mediator of oncogenic proteins and regulates many metastatic signaling molecules [24]. Elevated NEDD9 expression has been reported in many cancer types and is typically associated with tumor growth and invasion [19, 25, 26]. Data from Gabbasov et al [19]. showed that cancer cell-intrinsic NEDD9 expression promoted ovarian carcinoma development and invasion via induction of genes associated with oncogenic signaling and cancer stem cell properties (ALDH1a1 and ALDH1a2). Another study revealed that reduced expression of miR-451 increased chemoresistance in patients with metastatic castration-resistant prostate cancer by targeting NEDD9 [25]. Our work and other previous studies [13, 27, 28] have determined the role of NEDD9 in PAC. We show that high expression of NEDD9 is significantly correlated with clinical staging, lymph node metastasis, histologic stage, and significantly shorter survival time. [13] Interestingly, other studies have reported that NEDD9 acts as a tumor suppressor in breast cancer [29] and chronic myelogenous leukemia (CML) [30]. Previous research from Minn et al. found that downregulation of NEDD9 was part of a gene expression signature predicting breast cancer cell metastasis to the lung [29]. In a p210 *Bcr*/*Abl* mouse model of CML, knockdown of NEDD9 promoted the development, infiltration of myeloid cells in several tissues in CML [30]. In the current research, silencing of NEDD9 decreased miR-1252-5p loss-of-function-induced proliferation and migration of PAC cells, while ectopic expression of NEDD9 rescued miR-1252-5p overexpression-inhibited aggressive behavior of PAC cells, suggesting that miR-1252-5p inhibits NEDD9-mediated PAC progression.

Studies have shown that NEDD9 exerts its oncogenic function by enhancing activation of the SRC and STAT3 signaling pathways. [17–19] Employing genetically engineered mouse models (targeted disruption of *Nedd9, Nedd9^−/−^* genotype) in ovarian carcinoma, Gabbasov et al. showed that mice with the *Nedd9^−/−^* genotype exhibited decreased tumor growth and incidence of ascites via reduced expression and activation of signaling proteins, including SRC/STAT3 [19]. SRC, a 60-kDa member of the non-receptor tyrosine kinase family, is upregulated in 70% of pancreatic tumors [5, 9]. Inhibition of SRC signaling may retard pancreatic tumor growth and enhance gemcitabine cytotoxicity in xenografts of pancreatic cancer in mouse models [31]. In the current study, we examined whether SRC/STAT3 signaling was essential for the effect of miR-1252-5p/NEDD9 on PAC progression. We found that ectopic expression of NEDD9 rescued the inhibited SRC/STAT3 phosphorylation caused by miR-1252-5p overexpression, while the opposite results were observed in NEDD9- and miR-1252-5p-silenced cells. Further functional assays (MTT, wound healing, and western blotting) showed that PP1 substantially inhibited NEDD9-stimulated PAC progression. These data demonstrate that miR-1252-5p inhibits NEDD9-mediated biological behavior of PAC via the SRC/STAT3 pathway.

Subsequently, we explored the underlying mechanism that contributed to the downregulation of miR-1252-5p in PAC. It has been shown that the interactions between transcription factors and miRNAs are critical in many pathologic conditions. The *MYB* proto-oncogene has been widely accepted to act as a crucial oncogenic driver of many cancer types, including PAC [32]. Our results uncovered two putative biding sites of Myb in the region upstream of miR-1252 locus. The subsequent ChIP and luciferase assays demonstrated that Myb could negatively regulate miR-1252-5p expression by directly binding its promoter. Our data indicated that Myb could inhibit miR-1252 expression at the transcription level.

## CONCLUSIONS

To conclude, we demonstrate for the first time that miR-1252-5p expression is reduced in human PAC tissues and cell lines and that the reduced expression is associated with malignant clinicopathological features and poor prognosis of PAC patients. In addition, miR-1252-5p inhibits the proliferation, migration, invasion, and EMT of PAC cells by directly targeting NEDD9-mediation of SRC/STAT3 signaling. Our study also demonstrated that miR-1252-5p is inhibited by Myb at the transcription level (Figure 9F).

## Supplementary Material

Supplementary Methods

Supplementary Figures

Supplementary Table 1
