# Supplementary material for: MicroRNA-1252-5p, regulated by Myb, inhibits invasion and epithelial-mesenchymal transition of pancreatic cancer cells by targeting NEDD9
Source: Aging (Albany NY). 2021 Jul 27;13(14):18924–45. doi: 10.18632/aging.203344 (PMC8351675; doi:10.18632/aging.203344)
Supplement: Supplementary Methods [file aging-13-203344-s001.pdf]

SUPPLEMENTARY METHODS

Cell proliferation

We used 3-(4,5-dimethylthiazole-2-yl)-2,5-diphenyl tetrazolium bromide (MTT) assay and CellTiter-Glo luminescent cell viability kit (Promega Corporation, Madison, WI, USA) to examine cell proliferation. For MTT assay, PAC cell lines (2.0×10^3 cells per well) were cultured in a 96-well plate after transfection. An enzyme-linked immunosorbent assay (ELISA) plate reader was then used to detect the absorbance of 450 nm every 24 h for 4 days. For CellTiter-Glo luminescent cell viability assay, the transfected PAC cells were seeded in 96-well plates at a density of 1×10^4 cells/well in culture medium and cultured overnight at 37 ˚C. Following this, 100 µl CellTiter-Glo solution (Promega Corporation, Madison, WI, USA), mixed with the culture medium, were incubated for 20 min at room temperature, and the intensity of luminescence was detected.

Wound healing assay

Wound healing assay was performed as previously described [1]. Cells were grown to 90% confluence in a 6-well plate. A wound was made in the middle of the well using a 20-µl pipette tip. The cells were incubated in a medium containing 2% serum for 24 h. The wound closure was visualized by time-lapse imaging using a phase-contrast microscope (Nikon, Fukok, Japan) coupled to a CCD camera. Images of three selected fields were acquired for 0 h and 24 h. All experiments were performed in triplicate.

Transwell assay

Migration (without Matrigel) and invasion (with Matrigel) assays were conducted using pre-heated transwell chamber in 24-well plates (8-µm pores; Millipore Corp, Billerica, MA, USA). Matrigel and DMEM were mixed at a ratio of 1:5. A total of 5×10^4 cells were suspended in the upper chamber in 150 µL serum-free medium, and 600 µL DMEM containing 20% FBS was placed in the lower chamber. Cells were incubated at 37° C for 24h for migration assays and 16h for invasion assays. The cells in the apical membrane were gently removed using a cotton swab. Cells that migrated/invaded through to the under-side of the membrane were fixed using pre-cold paraformaldehyde for 30 min, stained with 0.5% crystal violet in 20% methanol rinsed under running water. The migrated/invaded cells were counted under an inverted microscope (Olympus Optical Co., Tokyo, Japan) in 5 random fields of view.

Luciferase reporter assay

Target genes of miR-1252-5p were screened for with the Starbase 3.0 program (http://starbase.sysu.edu.cn/). A luciferase reporter assay was then conducted to evaluate whether NEDD9 was a direct target gene of miR-1252-5p. Luciferase reporter plasmids predicted to interact with miR-1252-5p (pmiR-GLO-WT-NEDD9–3′UTR), with a corresponding mutated sequence (pmiR-GLO-MUT-NEDD9–3′UTR), were synthesized and purified by GenePharma. Panc-1 and BxPC-3 cells were plated into a 24-well plate and transfected using Lipofectamine 2000 (Invitrogen, USA). After 48 h, cells were collected, and luciferase activities were examined using the Dual-Luciferase Reporter Assay System (Promega, Madison, WI, USA), according to the manufacturer's protocol. *Renilla* luciferase activity was used to normalize the corresponding firefly luciferase activity.

Chromatin immunoprecipitation (ChIP) assay

We performed the CHIP assay by a ChIP assay kit (Millipore Corp, Billerica, MA, USA) as described earlier [2]. Briefly, cells were cross-linked with 1% formaldehyde solution for 10 min at room temperature. Cells then were neutralized with glycine (125 mM) and ultrasonicated (10s on, 10s off, 15 pulses) to obtain chromatin fragments of 200–500 base pairs. After centrifugation at 13,000 rpm, the supernatant was collected and transferred to three tubes containing the positive control antibody, the negative control IgG antibody, the target protein Myb specific antibody, respectively. Then, the endogenous DNA-protein complex was coupled to Protein A beads, and crosslinks were reversed (65° C for 16 h). The precipitated DNA was purified, followed by Proteinase K and RNase A treatment. After the non-specific DNA-protein complex was rinsed with the lysis buffer four times, the enrichment of DNA fragments was separated by SDS-PAGE and measured using the PCR assay.

Animal experiments

The animal experiments were approved by the Institutional Animal Care and Use Committee of Affiliated Hospital of Jiangnan University. The female BALB/c nude mice (5-week-old) were fed under standard conditions. The BxPC 3 cells (2×10^6) were injected subcutaneously into the mice's posterior flank. Tumor size was measured every 5 days, and was calculated using the formula (volume = length × width^2/2). When tumor volumes reached 100-150 mm^3,

agomiR NC or agomiR 1252-5p was directly injected into the tumors twice weekly.

Statistical analyses

All statistical analyses were performed using the PASW Statistics software program (version 19; SPSS, Chicago, IL, USA) and GraphPad Prism (version 5; GraphPad Inc., La Jolla, CA, USA). A two-tailed p-value < 0.05 was considered to be significant. Data are presented as the mean ± standard deviation. Significant differences were analyzed using paired student's t-test or one-way analysis of variance (one-way ANOVA) with Tukey's post hoc test. Spearman correlation test was conducted to analyze the association between miR-1252-5p and NEDD9 expression of PAC tissues. The Kaplan–Meier method and the log-rank test were performed to analyze the OS. Cox's hazards regression model was conducted to evaluate independent prognostic factors.

SUPPLEMENTARY REFERENCES

1. Mittal S, Kamath A, Joseph AM, Rajala MS. PLCγ1-dependent invasion and migration of cells expressing NSCLC-associated EGFR mutants. Int J Oncol. 2020; 57:989–1000. https://doi.org/10.3892/ijo.2020.5112 PMID:32945365

2. Parashar D, Geethadevi A, Aure MR, Mishra J, George J, Chen C, Mishra MK, Tahiri A, Zhao W, Nair B, Lu Y, Mangala LS, Rodriguez-Aguayo C, et al. miRNA551b-3p Activates an Oncostatin Signaling Module for the Progression of Triple-Negative Breast Cancer. Cell Rep. 2019; 29:4389–406.e10. https://doi.org/10.1016/j.celrep.2019.11.085 PMID:31875548
