# Supplementary material for: MicroRNA-1252-5p, regulated by Myb, inhibits invasion and epithelial-mesenchymal transition of pancreatic cancer cells by targeting NEDD9
Source: Aging (Albany NY). 2021 Jul 27;13(14):18924–45. doi: 10.18632/aging.203344 (PMC8351675; doi:10.18632/aging.203344)
Supplement: Supplementary Figures [file aging-13-203344-s002.pdf]

SUPPLEMENTARY FIGURES

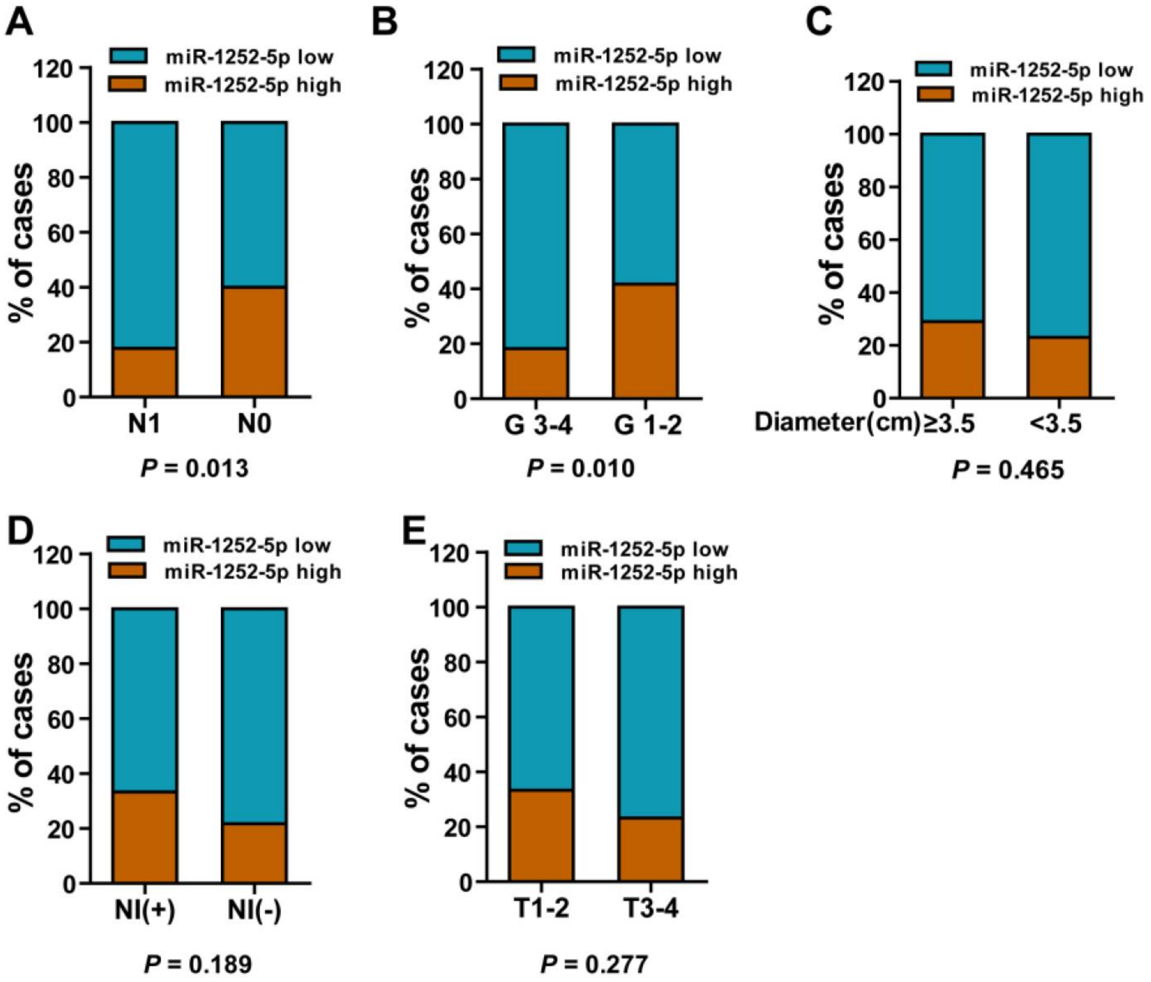

Supplementary Figure 1. Reduced miR-1252-5p expression via *in situ* hybridization in PACs was related to aggressive histologic characteristics and dismal prognosis. Expression of miR-1252-5p was significantly associated with (**A**) node invasion (*P* = 0.013), (**B**) high histologic grade (*P* = 0.010), but not with (**C**) tumor diameter (*P* = 0.465), (**D**) neural invasion (*P* = 0.189) and (**E**) T stage (*P* = 0.277). PAC, pancreatic cancer.

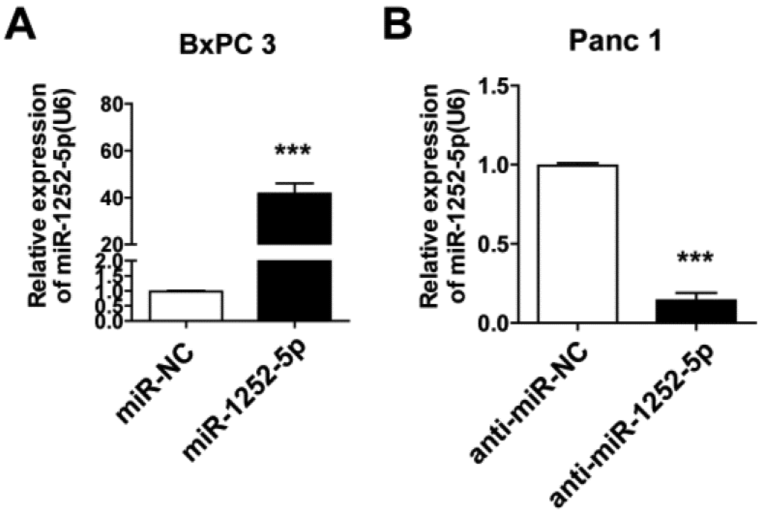

Supplementary Figure 2. qRT-PCR assay analysis of miR-1252-5p levels in BxPC 3 (**A**) and Panc-1 (**B**) cells transfected with miRNA vectors and corresponding negative control (NC). ***, $P < 0.001$.

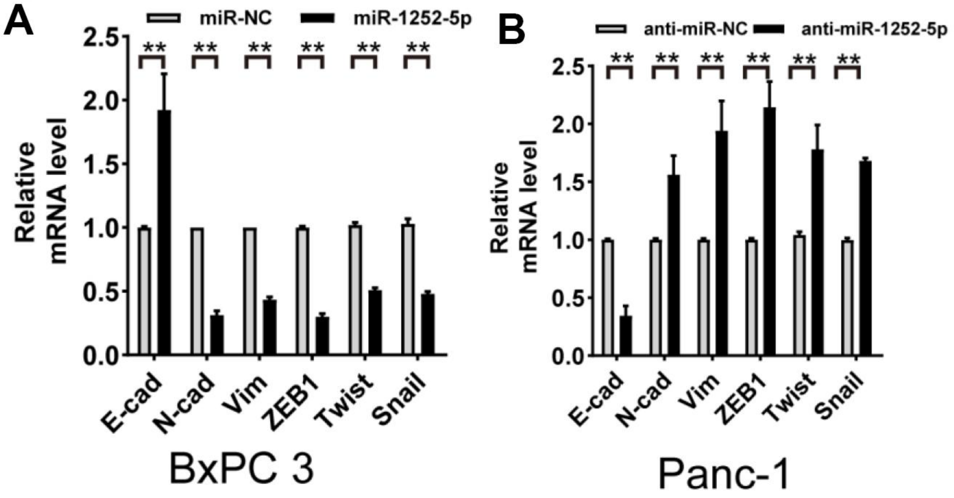

Supplementary Figure 3. qRT-PCR assays of the expression of epithelial-mesenchymal transition markers (E-cad, N-cad, Vim, ZEB1, Twist, and Snail) in BxPC 3 (**A**) and Panc-1 (**B**) cells after transfection with miRNA vectors and corresponding negative control (NC). GAPDH served as a loading control. NC, negative control; E-cad, E-cadherin; N-cad, N-cadherin; Vim, Vimentin. **, $P < 0.01$.

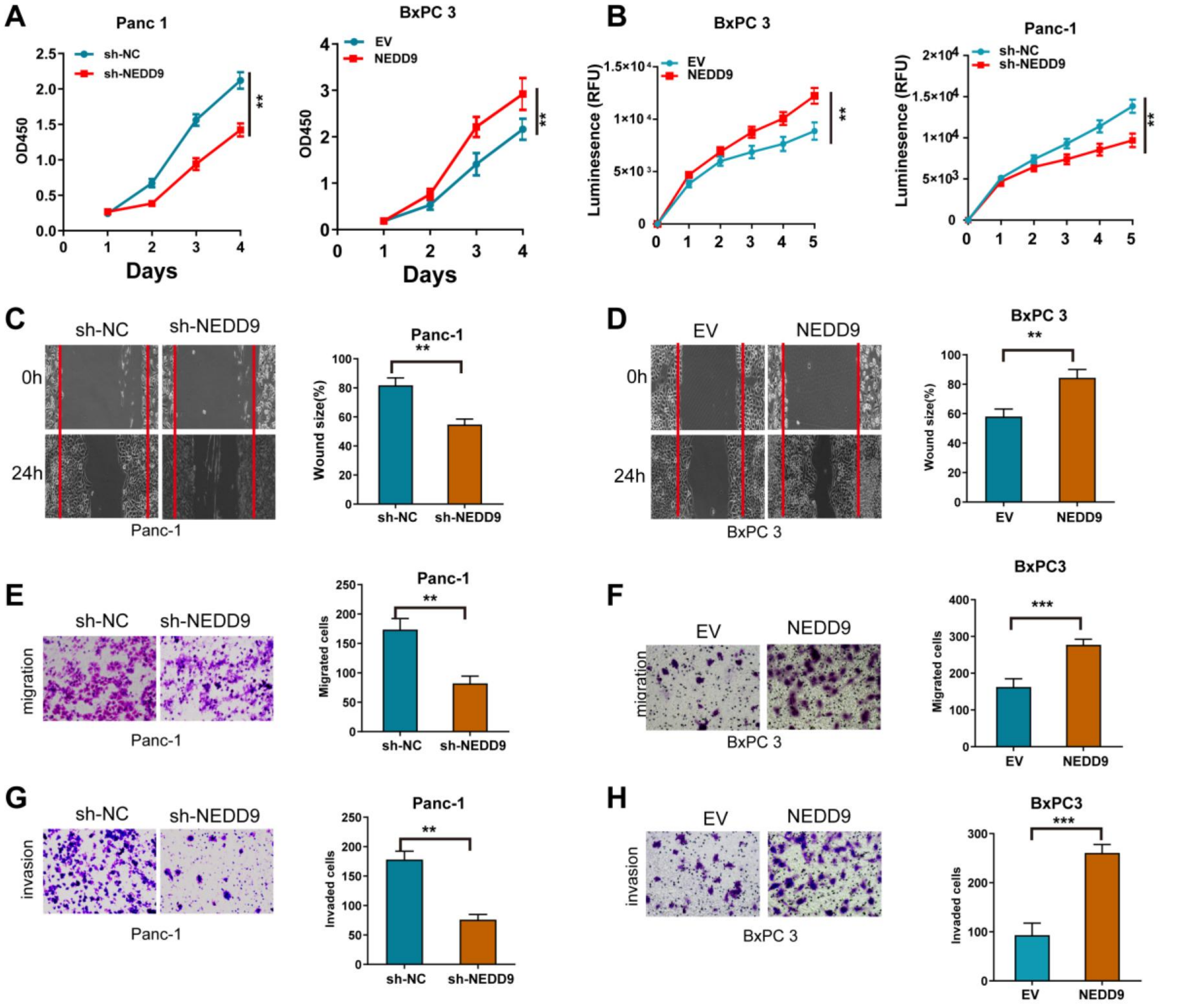

Supplementary Figure 4. NEDD9 promoted pancreatic cancer cells malignant behaviors. MTT (**A**), CellTiter-Glo luminescent cell viability (**B**), wound healing (**C**, **D**), and transwell (**E**–**H**) assays of cell proliferation, migration, and invasion in Panc-1 and BxPC 3 cells transfection with the indicated vectors. sh-NC and EV were used as the control. NC, negative control; EV, empty vector. $^{**}P < 0.01$; $^{***}P < 0.001$.
