# Supplementary material for: MicroRNA-1252-5p, regulated by Myb, inhibits invasion and epithelial-mesenchymal transition of pancreatic cancer cells by targeting NEDD9
Source: Aging (Albany NY). 2021 Jul 27;13(14):18924–45. doi: 10.18632/aging.203344 (PMC8351675; doi:10.18632/aging.203344)
Supplement: Supplementary Table 1 [file aging-13-203344-s003.pdf]

SUPPLEMENTARY TABLE

Supplementary Table 1. The sequences of the primers for QRT-PCR.

	Forward premier (5'-3')	Reverse premier (5'-3')
GAPDH	AGGTGAAGGTCGGAGTCAACG	GCTCCTGGAAGATGGTGATGG
ZEB1	TTACACCTTTGCATACAGAACCC	TTTACGATTACACCCAGACTGC
E-cadherin	CGAGAGCTACACGTTCACGG	GGGTGTCGAGGGAAAAATAGG
Vimentin	GCCCTAGACGAACTGGGTC	GGCTGCAACTGCCTAATGAG
N-cadherin	TTTGATGGAGGTCTCCTAACACC	ACGTTTAACACGTTGGAAATGTG
NEDD9	GACCGTCATAGAGCAGAACAC	TGCATGGGACCAATCAGAAGC
Twist	GTCCGCAGTCTTACGAGGAG	GCTTGAGGGTCTGAATCTTGCT
Snail	CTCTAGGCCCTGGCTGCTAC	GCCTGGCACTGGTACTTCTT
U6	TGACACGCAAATTCGTGAAGCGTTC	CCAGTCTCAGGGTCCGAGGTATTC
